# Characterization and Authentication of “Ricotta” Whey Cheeses through GC-FID Analysis of Fatty Acid Profile and Chemometrics

**DOI:** 10.3390/molecules27217401

**Published:** 2022-10-31

**Authors:** Alessandra Biancolillo, Samantha Reale, Martina Foschi, Emanuele Bertini, Lavinia Antonelli, Angelo Antonio D’Archivio

**Affiliations:** Department of Physical and Chemical Sciences, University of L’Aquila, 67100 L’Aquila, Italy

**Keywords:** fatty acid profiles, GC-FID, classification, ricotta cheese, dairies, sequential preprocessing through orthogonalization (SPORT), soft independent modelling by class analogy (SIMCA), PDO whey cheese, animal origin

## Abstract

The fatty acid (FA) profiles of 240 samples of ricotta whey cheese made from sheep, goat, cow, or water buffalo milk were analyzed by gas-chromatography (GC). Then, sequential preprocessing through orthogonalization (SPORT) was used in order to classify samples according to the nature of the milk they were made from. This strategy achieved excellent results, correctly classifying 77 (out of 80) validation samples. Eventually, since 36 (over 114) sheep ricotta whey cheeses were PDO products, a second classification problem, finalizing the discrimination of PDO and Non-PDO dairies, was faced. In this case, two classifiers were used, SPORT and soft independent modelling by class analogy (SIMCA). Both approaches provided more than satisfying results; in fact, SPORT properly assigned 63 (of 65) test samples, whereas the SIMCA model accepted 14 PDO individuals over 15 (93.3% sensitivity) and correctly rejected all the other samples (100.0% specificity). In conclusion, all the tested approaches resulted as suitable for the two fixed purposes. Eventually, variable importance in projection (VIP) analysis was used to understand which FAs characterize the different categories of ricotta. Among the 22 analyzed compounds, about 10 are considered the most relevant for the solution of the investigated problems.

## 1. Introduction

Whey cheeses are obtained from whey, the liquid remaining after the enzymatic coagulation of milk in cheesemaking processes, and are produced in different parts of the world, such as Italy (ricotta), Portugal (requeijão), Spain (requesón), and Turkey (lor), according to traditional methods.

Ricotta (literally meaning “cooked again”), which is not considered a cheese by the Italian law but a different kind of dairy product, is obtained by heat-induced coagulation (at 85–90 °C) of whey proteins (albumin and globulin) after the addition of acidifying agents, usually lemon or vinegar [1,2]. This process generates a curd of modest consistency incorporating the fats that after floating are transferred to draining moulds, cooled, and finally packaged. Although ricotta can be ripened or smoked, it is usually marketed as a fresh product, boasting remarkable nutritional properties because of the high value of the whey proteins, rich in sulphur-containing amino acids, and the relatively low-fat content. Despite the heat treatment inactivates the microflora by promoting the coagulation of whey proteins, the high humidity, the low salt content, and the pH close to neutrality make ricotta a suitable substrate for post-process microbial contamination. As a result, the shelf-life of fresh ricotta is relatively short [3], generally 4–14 days from production at 4 °C, depending on the treatment applied after curd floating and the kind of packaging.

Ricotta can be produced by using whey resulting from bovine, water buffalo, ovine and caprine cheese manufacturing, as well as their blends. Nevertheless, the substitution or mixing of bovine milk in dairy products labelled as obtained from water buffalo, goat, or sheep milk, is reported to be one of the most common adulterations in the dairy industry [4], economically motivated by the low commercial value of cow milk and seasonality of the milk production for the other species. It must be remarked that intentional mislabelling regarding the kind of whey/milk, apart from being a commercial fraud, is also illegal for sanitary reasons as consumers may be allergic or intolerant to the whey/milk components of specific animal species. The extraction and purification of denatured whey proteins coupled with their separation by isoelectric focusing was proposed to assess the fraudulent addition of cow whey in water buffalo ricotta [5]. In addition, mass spectrometry-based analytical methods were developed to monitor species-specific peptides derived from whey proteins [6,7].

Among the different kinds of Italian ricotta, two products, “Ricotta di Bufala Campana”, produced with 100% water buffalo’s whey, and “Ricotta Romana”, obtained from 100% sheep’s whey, have been approved as Protected Denomination of Origin (PDO) specialties. Dairy products designed as PDO, because of their higher commercial value compared to ordinary products, are potential targets of fraudulent adulterations, including the above-mentioned mislabelling of whey origin. Although it is known that the kind of whey primarily affects the sensorial properties of the ricotta whey cheeses [2], several additional factors, such as the local know-how adopted in processing [8], the breed of dairy animals and their diet [9,10] contribute to conferring their own distinctive origin-related uniqueness to products designed as PDO. PDO Ricotta Romana, for instance, is obtained from full-fat sheep whey provided by the commonest breeds in the Lazio Region. Its distinctive sweetish flavour results from the type of feed given to the milk ewes, forage from natural pastures, meadows, and the characteristic grasslands of the production territory. It follows that the authentication of a PDO ricotta whey cheese cannot be limited to the identification of the whey origin, but PDO and non-PDO products obtained with the same kind of whey should be also discriminated. As discussed previously, different approaches have been proposed to detect the undeclared addition of cow whey to whey cheeses from different animal origins. Furthermore, a number of instrumental methods coupled to chemometric approaches have been used to address geographical traceability and authentication of many dairy products [11]. Nevertheless, neither analytical methods for the geographical traceability of ricotta whey cheeses, including PDO specialties, nor strategies for the discrimination of ricotta whey cheeses according to the whey origin have been reported in the literature to the best of our knowledge.

In light of these considerations, the purpose of the present work is manifold. First of all, the study focuses on the characterization of FAs in ricotta produced with different kinds of milk. For this purpose, FA profiles were determined by gas chromatography (GC) coupled to flame ionization detection (FID) applied to the FA methyl esters obtained by the trans-esterification of the glyceridic fraction extracted from ricotta whey cheeses.

A further goal of the present study is the creation of models that are able to solve two different problems: the classification of samples according to the animal origin and the discrimination between PDO and non-PDO ricotta samples. This work, in addition to representing a novelty concerning the literature is also aimed at protecting consumers. In fact, it represents a useful tool to recognize fraud attempts related to the sale of PDO ricotta instead of non-PDO cheese or the illicit mislabelling of the nature of the milk.

## 2. Results and Discussion

In this study, the fatty acid profiles of five types of ricotta whey cheeses are reported. Specifically, as detailed in Section 3.1, the analysed ricotta samples were obtained from sheep (PDO and Non-PDO), goat, cow, and water buffalo whey.

### 2.1. FA Profiles in Ricotta Cheese from Different Milks

Table 1 reports the mean relative peak area for each of the 22 FAs identified in the diverse ricotta whey cheeses under investigation. The observed FAs profiles quite closely reflect those of the related milks [12,13]. In all the analysed ricotta whey cheeses the most abundant FA is the saturated C16 fatty acid palmitic acid (#10), which reaches the greatest quantities (ca. 36% and 32%, respectively) in water buffalo and cow ricotta, while in sheep (even in the PDO Ricotta Romana) and goat ricotta accounts for about 25%. The second and third most abundant FAs are myristic acid (#7) and oleic acid (#15) which account for about 15% in all the ricotta samples analysed here.

As a general trend, the FA profiles of cow and water buffalo ricotta whey cheeses are more similar from each other compared to that of sheep and goat ones. For example, capric acid (#4) and caprylic acid (#3) content in the goat and sheep ricotta are about three and two times, respectively, that observed in cow and water buffalo ricotta. Incidentally, the higher content of C8:0 and C10:0 (#3 and #4) fatty acids in the milk and dairy products of sheep and goat in comparison to cow and water buffalo ones is responsible of the specific aroma of milk and dairy products obtained from milk of the former two ruminants [14,15]. Elaidic acid (#14) is also about two times more abundant in sheep and goat ricotta than in cow and water buffalo varieties. Elaidic acid C18:1-(9*E*) is the simplest trans FA and naturally occurs in ruminant fat, meat, and dairy products [16,17]. On the other hand, palmitoleic acid (#11) is about three times more abundant in cow and water buffalo ricotta than in sheep and goat ricotta. The essential FA linoleic acid (#16), belonging to the omega-6 class, has a relative abundance that barely exceeds 1% in all the five ricotta whey cheeses. With regard to the essential FAs, it is interesting to note that the content of alpha-linolenic acid (#18) in the PDO Ricotta Romana is about two times that of sheep and goat ricotta and even four times the content in cow and water buffalo ricotta.

The above findings can be explained by considering the taxonomic classification of the four ruminants, that although all belonging to the Bovidae family, are classified in two different subfamilies, water buffalo (*Bubalus bubalis*) and cow (*Bos taurus*) are assigned to the subfamily of Bovinae, whereas goat (*Capra hircus*) and sheep (*Ovis aries*) to that of Caprinae.

Along with animal taxonomy (also including the species and individual traits) other factors, such as the animal breeding and nutrition, the period of lactation, and the type of production process, can affect the FA profiles of dairy products [12]. Most of these factors are related to the geographical origin and can be responsible for the subtle differences in the FA profile of Ricotta Romana PDO compared to that of the other Non-PDO sheep ricotta whey cheeses here analysed.

### 2.2. Classification of Ricotta Cheeses According to the Type of Whey

SPORT analysis was applied to the FA profiles to discriminate the different cheeses according to the nature of the milk they were made from. In order to allow external validation of the calibration model, samples were divided into a training and a test set of 160 and 80 types, respectively. The former set contains 74 samples belonging to the Class Sheep, 34 objects appertaining to the Class Cow, and 26 belonging to the Class Goat and Class Buffalo (each), whereas the test set comprised 40 samples made from sheep milk, 20 from cow milk, 10 belonging to Class Goat and 10 to Class Buffalo.

Due to the nature of the data, SPORT was exploited to ensemble two pretreatments: mean-centring and autoscaling. Data were preprocessed in the aforementioned order; consequently, the first modelled block (**X**_1_) is the one including the mean-centred profiles, while the second one (**X**_2_) is the autoscaled data block. Model parameters and the correct classification rate in cross-validation (CCRcv (%)) are reported in Table 2.

**Figure 1:**
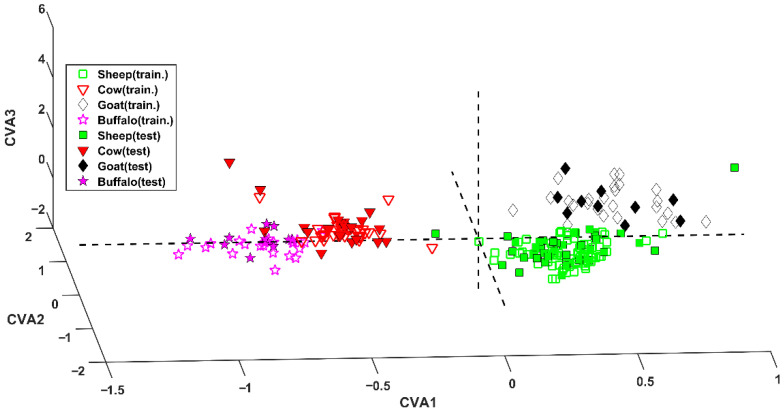
SPORT analysis. Projection of samples onto the space defined by the canonical variates (CVAs).

The application of the model to the test set led to the correct prediction of 77 samples (out of 80), achieving a correct classification rate of 96.2%. The excellent results can also be graphically observed in Figure 1, where samples are projected onto the space defined by the three canonical variates (CVAs) [18].

Inspecting this representation, it is evident the first CVA discriminates between *bovinae* and *caprinae*. In fact, sheep and goat samples (green squares and black diamonds, respectively) fall at positive values of this component, whereas cow and water buffalo cheeses (red downward triangles and magenta stars, respectively) present negative values of CVA1. Within the two subfamilies (Bovinae and Caprinae) the genus can be discerned by means of the second and the third canonical variates. In particular, cow and goat samples fall at positive values of CVA3, contrarily to samples appertaining to the other two classes.

**Figure 2:**
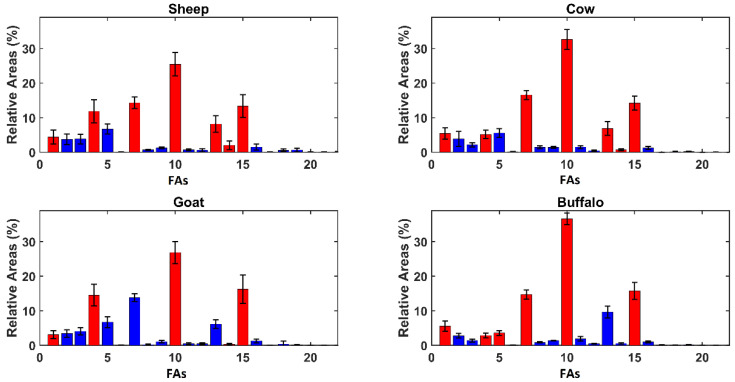
Average Relative Areas per genus. Red bars represent variables presenting a VIP index higher than 1 and blue bars represent the other variables. The compounds’ enumeration is the same used in Table 1.

In order to give a deeper insight into the model, variable importance in projection (VIP) analysis [19] was coupled with SPORT, as suggested in the literature [20]. The results of VIP analysis are displayed in Figure 2, showing the FA mean relative areas observed in the different kinds of ricotta whey cheese.

As discussed previously, Figure 2 reveals evident differences in terms of the relative areas among the samples produced from animals of different genus, but the FA profiles are similar between animals of the same subfamily. The inspection of VIP figures has revealed four compounds that have been highlighted as relevant for each category: butanoic acid (#1), capric acid (#4), palmitic acid (#10), and oleic acid (#15). Other relevant compounds are myristic acid (#7) and elaidic acid (#14), selected in each class except for goat and water buffalo, respectively. The most different selection is given by water buffalo ricotta, where lauric acid (#5) also appears significant.

### 2.3. Classification of PDO and Non-PDO Ricotta Cheeses

Eventually, the possibility of discriminating PDO ricotta from all the other samples has been tested. Initially, classification was achieved by SPORT. Subsequently, given the asymmetrical nature of the classification problem, a class-modelling approach was also exploited.

Similarly, as before, the SPORT model finalized for the discrimination of PDO and Non-PDO ricotta was created. The preprocessing approaches used were mean-centring and autoscaling (applied in this order: i.e., the first modelled block was pretreated by mean-centring, whereas the second one was autoscaled). In this case, the model providing the best compromise between a parsimonious complexity and high CCRcv was the one extracting only 7 LVs from the mean-centred block. This led to a CCRcv of 100.0% for Class PDO and of 98.6% for Class Non-PDO. The application of this model to the validation set allowed the correct prediction of all the PDO samples and of 63 (over 65) Non-PDO objects (corresponding to a correct classification rate of 96.9% for this category). This represents a relevant achievement because it demonstrates the efficiency of the model at recognizing PDO sheep ricotta samples despite the Non-PDO class containing sheep ricotta types. This accomplishment is noteworthy because it confirms that there is an actual significant difference in terms of FA profiles between PDO and non-PDO whey cheeses. Additionally, in this case, VIP analysis was used to investigate which compounds characterize PDO/Non-PDO samples. This further inspection revealed that the same seven compounds are relevant for both classes. In particular, butanoic acid (#1), capric acid (#4), myristic acid (#7), palmitic acid (#10), stearic acid (#13), elaidic acid (#14), and oleic acid (#15) exhibit significantly different relative abundances in these two categories.

Despite the excellent results obtained, SIMCA was also used for modelling the PDO-class. In this case, the two different pretreatments were separately tested. Consequently, two SIMCA models were built—sensitivity, specificity, and efficiency (obtained in a seven-fold cross-validation procedure)—as shown in Table 3.

**Figure 3:**
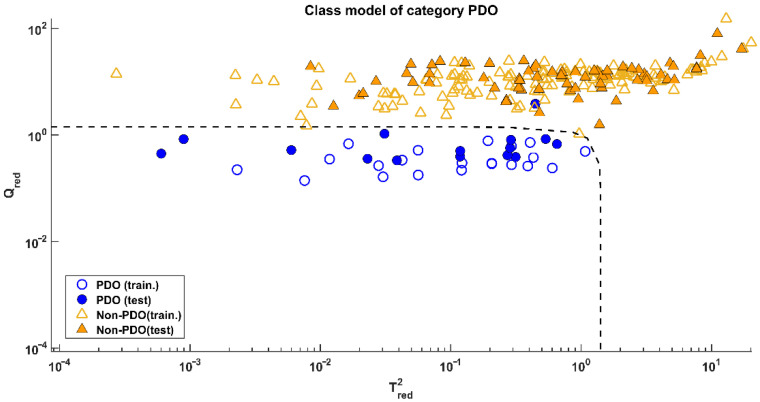
SIMCA: Projection of the samples onto the $Q_{\mathrm{red}}\;\mathrm{vs}.\;T_{\mathrm{red}}^{2}$ model space of class PDO.

Since both models provided the same results, the one calculated on data processed by the mildest pretreatment (mean-centring) was preferred. Its application on the test set led to the results visible in Figure 3, corresponding to a sensitivity of 93.3% (only one misclassified test sample over 15) and a specificity of 100%.

## 3. Materials and Methods

### 3.1. Ricotta Samples

A total of 240 ricotta cheese whey samples from different batches prepared by 15 different manufacturers were analysed by GC/FID, as described in Section 3.2. A number of 114 were made from sheep milk, (of these, 78 were Non-PDO and 36 PDO), 36 from goat milk, 54 from cow milk, and 36 from water buffalo milk).

### 3.2. Fatty Acid Extraction, FAME Preparation, and GC-FID Analyis

Fatty acid (FA) contents in Ricotta cheeses were determined after modified fat extraction according to the method described by de Jong and Badings [21]. Ricotta cheese was maintained at 120 °C in the oven for 30 min to remove some of its water to facilitate the following extraction step. Of partially dehydrated ricotta cheese, 1 g was mixed with 2 g of anhydrous sodium sulphate (Na_2_SO_4_) and 0.3 mL of 2.5 M sulfuric acid solution and then extracted three times with 3 mL ether/heptane (1:1, v/v) from Sigma-Aldrich (St. Louis, MO, USA). Each time the solution was clarified by 5 min of centrifugation at 1500 rpm, and the upper solvent layer was added with 1 g of anhydrous Na_2_SO_4_ to adsorb residual water.

Fatty acid methyl esters (FAMEs) were prepared by fast trans-methylation conducted under alkali-catalysed conditions with potassium hydroxide according to the International Organization for Standardization [22]. Briefly, the elute obtained was added with 200 μL of 2 M methanolic potassium hydroxide solution and the glass vial was shaken vigorously for about 30 s. After an initial cloudiness due to the separation of glycerol, the solution became clear. About 1 g of sodium hydrogen sulfate monohydrate was added to neutralize the potassium hydroxide excess and prevent saponification of the methyl esters. The solution was shaken briefly and, after the salt had settled, the upper layer was transferred into a vial. The obtained solution was suitable for GC analysis.

FAME profile was determined using a Thermo Trace GC Ultra 7820 gas chromatograph system (Thermo Fisher Scientific, Waltham, MA, US) equipped with a flame ionization detector (FID). The separation was achieved using a 30 m × 0.25 mm × 0.25μm fused silica capillary column SP^TM^-2380 (Supelco, Bellafonte, PA, USA). At first, the GC oven temperature was maintained at 80 °C for 2 min, then the temperature was increased to 180 °C at a rate of 10 °C/min and held at 180 °C for 10 min. After that, the oven temperature was increased to 250 °C at a rate of 10 °C/min and finally held at 250 °C for 2 min. The SSL injector was kept at 250 °C throughout the whole analysis and injections were performed in split mode with a 25 mL/min split flow. The detector temperature was maintained at 250 °C with 350 mL/min of air and 40 mL/min of hydrogen. Hydrogen was constantly supplied by the GENius PF500 (FullTech Instruments, Rome, Italy) generator and used as carrier gas at a constant pressure of 0.5 bar.

The mixtures of fatty acid methyl esters (FAMEs) Grain FAME Mix (Supelco, Bellafonte, PA, USA) and C4-C24 even carbon-saturated FAMEs (Supelco, Bellafonte, PA, USA) were used in the GC analysis to identify the peaks by comparing the retention times. Peak areas of the 22 identified FAMEs were quantified by Xcalibur^TM^ software (Thermo Fisher Scientific, Waltham, MA, USA) and the contents of each FA in the samples were expressed as the percentage ratio of the peak area of the related FAME over the sum of the 22 identified FAME peak areas.

### 3.3. Chemometric Tools

In the literature, several chemometric classification methods are available [23]. In general, they can be divided into two categories: the discriminant approaches and the class-modelling strategies. The main difference between these two families of methods is associated with the way the two divide the multi-dimensional space of the samples [24]. In one case (the discriminant one) the space is divided into as many regions as the number of classes in the training set. However, when applying class-modelling approaches, each category is individually modelled. Given their different natures, there are situations in which class-modelling strategies are more suitable than discriminant methods and vice versa [23]. In particular, the former methods are especially useful for asymmetric classification, i.e., in those cases where the interest is mainly on a specific category. In the present work, two classifiers have been applied: SPORT (belonging to the discriminant methods family) [25] and SIMCA (a class-modelling approach) [26]. The former approach is a sequential method for ensemble preprocessing derived by sequential and orthogonalized partial least squares (SO-PLS) [27] coupled with linear discriminant analysis (LDA) [28] in the same fashion as suggested for SO-PLS [29]. When testing the two preprocessing methods (as in the present work), the creation of a SPORT model can be achieved as follows:

the **X** data block is separately preprocessed by the two pretreatments, obtaining two diverse predictor blocks: **X_1_** and **X_2_**.

A **Y** dummy [30] encoding the class-membership is fitted to **X_1_** by PLS.

**X_2_** is orthogonalized with respect to the **X**-scores extracted in (2), obtaining **X_2,orth._**

**X_2,orth_** is used to predict the **Y**-residuals from (1).

The final model is estimated summing up the contribution from (1) and (4).

Eventually, LDA can be applied on the **Y** predicted by the SPORT model as described in [25,31].

In the present work, SPORT has been used to solve two different classification problems; one, it was utilized for the classification of samples according to the milk the dairies are made from (the four-classes problem: Sheep, Goat, Cow, and Water buffalo); and second, for the discrimination of PDO and Non-PDO sheep ricottas.

SIMCA is a class-modelling approach which allows the individual estimation of the class-regions of specific categories of interest. Here, it was used to recognize PDO-ricotta samples with respect to all the other investigated dairies.

SIMCA starts from the calculation of a principal component model (PCA) [32] on the training samples belonging to the class of interest. Eventually, the distance (d) between each sample and the class-model is estimated as formulated in Equation (1):

(1) $$d=\sqrt{{\left(T_{0.95}^{2}\right)}^{2}+{\left(Q_{0.95}\right)}^{2}}$$

where 𝑇^2^ is the squared Mahalanobis distance from the centre of the scores space and 𝑄 is the sum of squared residuals. Both entities are normalized by the 95th percentile of their corresponding distributions [33,34].

Eventually, samples were accepted or rejected according to the dimension of d. Customarily, the acceptance threshold is $\sqrt{2}$; consequently, when d is smaller than this value, the object is accepted by the model (and, therefore, predicted as belonging to the modelled class); otherwise, it is rejected.

Due to the nature of SIMCA, results are often provided in terms of sensitivity, specificity, and efficiency. Sensitivity represents the percentage of samples correctly accepted by the model, whereas specificity is the percentage of samples properly rejected. The efficiency is their geometric average, and it is here exploited in order to define model parameters (e.g., number of principal components or pretreatment).

## 4. Conclusions

This work was tailored to address different goals. First of all, the aim was to delineate the FA profiles of ricotta cheeses produced from the whey of four different animals.

Subsequently, data were analysed from a more chemometric-oriented standpoint, and two different classification problems were faced. The first one aimed at classifying the ricotta samples on the basis of the milk they were made from, whereas the second one was utilized for discriminating the sheep “Ricotta Romana” PDO from all other dairy products.

The discrimination between PDO “Ricotta Romana” and all the others was carried out by SPORT and SIMCA. Both strategies were suitable for the purpose. SPORT properly classified ~97% of test samples, whereas SIMCA achieved a sensitivity of 93.3% and a specificity of 100%. These accomplishments are particularly interesting because, considering the presence of Non-PDO sheep ricotta samples in the calibration and in the test set, it indicates a significant difference between PDO and Non-PDO whey cheeses, which has never been documented before in the literature to the best of our knowledge.

## Figures and Tables

**Table 1:** FA profiles of ricotta whey cheeses. Peak number (#); RT (min): Observed retention time, FA common name and abbreviation and mean relative (A%) peak area in the GC/FID chromatograms with related standard error (SE).

#	RT (min)	FA	Abbreviation	Sheep (Non-PDO) *n* = 78	Sheep (Ricotta Romana PDO) *n* = 36	Goat *n* = 36	Cow *n* = 54	Water Buffalo *n* = 36
				Mean A% ± SE	Mean A% ± SE	Mean A% ± SE	Mean A% ± SE	Mean A% ± SE
1	2.55	butanoic acid	C4:0	4.94 ± 0.26	3.25 ± 0.10	3.16 ± 0.19	5.69 ± 0.27	5.56 ± 0.25
2	3.65	caproic acid	C6:0	4.10 ± 0.19	2.83 ± 0.03	3.47 ± 0.18	3.90 ± 0.29	3.29 ± 0.47
3	5.51	caprylic acid	C8:0	4.09 ± 0.17	3.07 ± 0.04	4.11 ± 0.17	2.16 ± 0.08	1.41 ± 0.07
4	7.65	capric acid	C10:0	12.67 ± 0.42	9.75 ± 0.15	14.60 ± 0.52	5.19 ± 0.16	2.87 ± 0.11
5	9.67	lauric acid	C12:0	7.10 ± 0.19	5.66 ± 0.09	6.76 ± 0.27	5.59 ± 0.18	3.59 ± 0.11
6	10.60	tridecanoic acid	C13:0	0.06 ± 0.01	0.05 ± 0.01	0.07 ± 0.01	0.11 ± 0.01	0.09 ± 0.01
7	11.50	myristic acid	C14:0	15.45 ± 0.55	13.02 ± 0.08	13.91 ± 0.19	16.65 ± 0.20	14.70 ± 0.23
8	12.12	myristoleic acid	C14:1-(9*Z*)	0.63 ± 0.02	0.75 ± 0.02	0.23 ± 0.03	1.45 ± 0.06	0.87 ± 0.03
9	12.35	pentadecanoic acid	C15:0	1.24 ± 0.03	1.30 ± 0.02	1.10 ± 0.06	1.42 ± 0.03	1.42 ± 0.02
10	13.31	palmitic acid	C16:0	25.06 ± 0.49	26.17 ± 0.14	26.93 ± 0.54	32.77 ± 0.39	36.47 ± 0.31
11	13.91	palmitoleic acid	C16:1-(9*Z*)	0.70 ± 0.03	0.69 ± 0.03	0.54 ± 0.05	1.48 ± 0.06	1.91 ± 0.11
12	14.30	margaric acid	C17:0	0.52 ± 0.02	0.73 ± 0.13	0.51 ± 0.04	0.44 ± 0.02	0.52 ± 0.02
13	15.55	stearic acid	C18:0	7.32 ± 0.26	9.89 ± 0.21	6.18 ± 0.21	6.92 ± 0.27	9.64 ± 0.28
14	16.09	elaidic acid	C18:1-(9*E*)	1.23 ± 0.08	3.61 ± 0.05	0.31 ± 0.04	1.26 ± 0.39	0.59 ± 0.04
15	16.32	oleic acid	C18:1-(9*Z*)	12.27 ± 0.40	15.49 ± 0.17	16.29 ± 0.68	13.45 ± 0.50	15.69 ± 0.41
16	17.68	linoleic acid	C18:2*-*(9*Z*,*12Z*)	1.68 ± 0.23	1.43 ± 0.03	1.30 ± 0.10	1.22 ± 0.06	1.01 ± 0.04
17	18.86	arachidic acid	C20:0	0.05 ± 0.01	0.06 ± 0.01	0.02 ± 0.01	n.d.	0.09 ± 0.01
18	19.72	alpha-linolenic acid	C18:3-(9*Z*,12*Z*,15*Z*)	0.45 ± 0.04	0.80 ± 0.04	0.38 ± 0.14	0.12 ± 0.03	0.08 ± 0.01
19	20.22	*cis*-11-eicosenoic acid	C20:1-(11*Z*)	0.40 ± 0.08	1.32 ± 0.05	0.12 ± 0.03	0.17 ± 0.02	0.16 ± 0.02
20	23.60	behenic acid	C22:0	0.016 ± 0.004	0.02 ± 0.01	n.d.	n.d.	0.017 ± 0.005
21	24.32	erucic acid	C22:1-(13*Z*)	0.03 ± 0.01	0.01 ± 0.01	0.02 ± 0.01	n.d.	0.04 ± 0.01
22	26.52	lignoceric acid	C24:0	0.02 ± 0.01	0.07 ± 0.05	n.d.	n.d.	0.008 ± 0.003

n.d. = not detected.

**Table 2:** SPORT analysis for classification of FA profiles according to the type of milk.

LVs (X_1_, X_2_)	CCRcv (%)			
	Class Sheep	Class Cow	Class Goat	Class Buffalo
4, 3	98.6	94.1	100.0	100.0

**Table 3:** SIMCA analysis of Class PDO Ricotta Romana.

Pretreatment	PCs	Sensitivity	Specificity	Efficiency
Mean-centring	1	85.7	99.7	92.4
Autoscaling	1	85.7	99.7	92.4

## Data Availability

The data presented in this study are available on request from the corresponding author.
